# [Corrigendum] Gemcitabine combined with an engineered oncolytic vaccinia virus exhibits a synergistic suppressive effect on the tumor growth of pancreatic cancer 

**DOI:** 10.3892/or.2025.9013

**Published:** 2025-10-30

**Authors:** Wanyuan Chen, Weimin Fan, Guoqing Ru, Fang Huang, Xiaming Lu, Xin Zhang, Xiaozhou Mou, Shibing Wang

Oncol Rep 41: 67–76, 2019; DOI: 10.3892/or.2018.6817

Following the publication of the above article, the authors drew to the Editor's attention that certain of the data in Fig. 6 on p. 74 had been assembled incorrectly. Specifically, the data panels showing the results of the SW1990/oVV/H&E and the SW1990/GEM/oVV/H&E experiments, and the SW1990/Gemcitabine/IHC-Smac and the SW1990/GEM/oVV/IHC-Smac experiments, respectively, contained overlapping sections, such that the data, which were intended to show the results of differently performed experiments, had been derived from the same original sources. Furthermore, upon performing an independent analysis of the data in the Editorial Office, it was noted that the data panels selected for the SW1990/oVV-Smac/TUNEL and the SW1990/Gemcitabine&oVV-Smac/TUNEL experiments also contained overlapping sections, and concerning the western blot data shown in Fig. 3B on p. 71, the Survivin protein bands were strikingly similar to data which had appeared in an earlier paper featuring some of the same authors in the journal *Scientific Reports*.

After having re-examined these figures, the authors realized that these additional cases of data duplication/re-use were also in need of correction. The revised versions of Figs. 3 and 6, now showing the correct data for the SW1990/GEM/oVV/H&E, SW1990/GEM/oVV/IHC-Smac and SW1990/oVV-Smac/TUNEL experiments in Fig. 3, and the correct Survivin western blot data in Fig. 6, are shown on the next page. The authors wish to emphasize that the errors made in terms of the assembly of the data in these figures did not affect the overall conclusions reported in the paper. The authors are grateful to the Editor of *Oncology Reports* for granting them this opportunity to publish a Corrigendum, and apologize to both the Editor and the readership for any inconvenience caused.

## Figures and Tables

**Figure 3.**
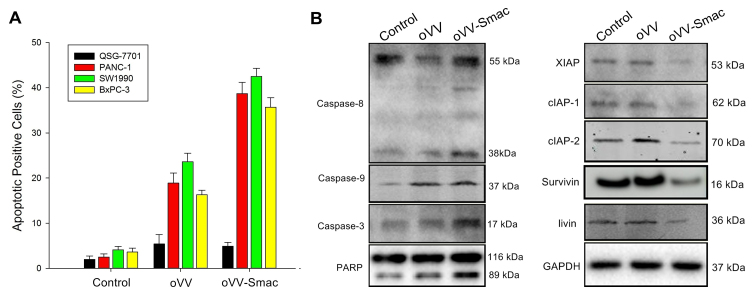
oVV-Smac induces apoptosis in pancreatic cancer cells *in vitro.* (A) Apoptosis analysis using Annexin V-FITC/PI double staining. SW1990 cells were infected with oVV or oVV-Smac (MOI=5) for 24 h. The florescence was analyzed by flow cytometry. Data are presented as mean ± SD of three separate experiments. **P<0.01, ***P<0.001. (B) SW1990 cells were infected with oVV-Smac or oVV (5 MOI) for 24 h. Whole cell extracts were prepared and immunoblotted for the detection of activation of caspase and IAP pathway. GAPDH was used as a loading control. oVV, oncolytic vaccinia virus; oVV-Smac, Smac-armed oncolytic vaccinia virus.

**Figure 6.**
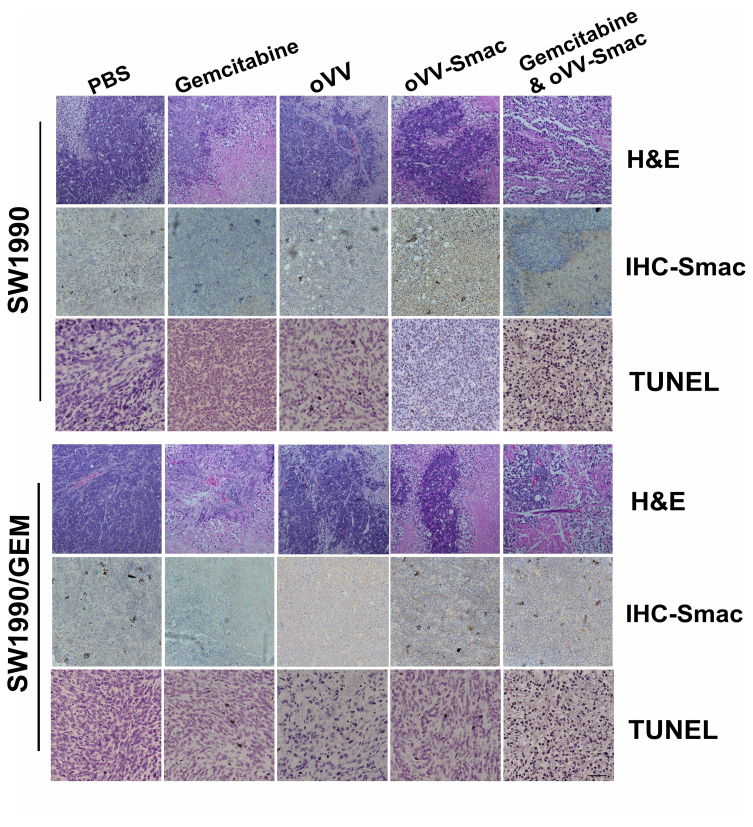
Histopathological analysis. Tumor samples from the different groups were subjected to H&E staining, immunohistochemical and TUNEL analysis. Magnification, ×200. oVV, oncolytic vaccinia virus; oVV-Smac, Smac-armed oncolytic vaccinia virus.

